# Neurochemical Differences in Spinocerebellar Ataxia Type 14 and 1

**DOI:** 10.1007/s12311-020-01201-y

**Published:** 2020-10-15

**Authors:** Anne Sophie Grosch, Jan Leo Rinnenthal, Maria Rönnefarth, Silke Lux, Michael Scheel, Matthias Endres, Alexander U. Brandt, Friedemann Paul, Tanja Schmitz-Hübsch, Martina Minnerop, Sarah Doss

**Affiliations:** 1 Department of Neurology, Charité University Medicine Berlin, Charitéplatz 1, 10117 Berlin, Germany; 2 Department of Pathology, SANA Klinikum Offenbach, Offenbach, Germany; 3 Experimental and Clinical Research Center, Max Delbrueck Center for Molecular Medicine and Charité University Medicine Berlin, Berlin, Germany; 4 NeuroCure Clinical Research Center, Charité University Medicine Berlin, Berlin, Germany; 5 Berlin Institute of Health (BIH), Anna-Louisa-Karsch-Str. 2, 10178 Berlin, Germany; 6 Department of Psychiatry and Psychotherapy, University Hospital Bonn, Bonn, Germany; 7 Department of Neuroradiology, Charité University Medicine Berlin, Berlin, Germany; 8 Center for Stroke Research Berlin (CSB), Charité University Medicine Berlin, Berlin, Germany; 9 German Center of Neurodegenerative Diseases (DZNE), Berlin, Germany; 10 German Centre for Cardiovascular Research (DZHK), Berlin, Germany; 11 Department of Neurology, University of California, Irvine, CA USA; 12 Institute of Neuroscience and Medicine (INM-1), Research Centre Juelich, Juelich, Germany; 13 Medical Faculty, Department of Neurology and Institute of Clinical Neuroscience and Medical Psychology, Center for Movement Disorders and Neuromodulation, Heinrich-Heine University, Düsseldorf, Germany; 14 Movement Disorders Section, Department of Neurological Sciences, University of Nebraska Medical Center, Omaha, NE USA

**Keywords:** Spinocerebellar ataxia, ^1^H magnetic resonance spectroscopy, SCA1, SCA14, Neurochemical profile

## Abstract

Autosomal-dominant spinocerebellar ataxias (SCA) are neurodegenerative diseases characterized by progressive ataxia. Here, we report on neurometabolic alterations in spinocerebellar ataxia type 1 (SCA1; SCA-ATXN1) and 14 (SCA14; SCA-PRKCG) assessed by non-invasive ^1^H magnetic resonance spectroscopy. Three Tesla ^1^H magnetic resonance spectroscopy was performed in 17 SCA14, 14 SCA1 patients, and in 31 healthy volunteers. We assessed metabolites in the cerebellar vermis, right cerebellar hemisphere, pons, prefrontal, and motor cortex. Additionally, clinical characteristics were obtained for each patient to correlate them with metabolites. In SCA14, metabolic changes were restricted to the cerebellar vermis compared with widespread neurochemical alterations in SCA1. In SCA14, total N-acetylaspartate (tNAA) was reduced in the vermis by 34%. In SCA1, tNAA was reduced in the vermis (24%), cerebellar hemisphere (26%), and pons (25%). SCA14 patients showed 24% lower glutamate+glutamine (Glx) and 46% lower γ-aminobutyric acid (GABA) in the vermis, while SCA1 patients showed no alterations in Glx and GABA. SCA1 revealed a decrease of aspartate (Asp) in the vermis (62%) and an elevation in the prefrontal cortex (130%) as well as an elevation of myo-inositol (Ins) in the cerebellar hemisphere (51%) and pons (46%). No changes of Asp and Ins were detected in SCA14. Beyond, glucose (Glc) was increased in the vermis of both SCA14 (155%) and SCA1 (247%). ^1^H magnetic resonance spectroscopy revealed differing neurochemical profiles in SCA1 and SCA14 and confirmed metabolic changes that may be indicative for neuronal loss and dysfunctional energy metabolism. Therefore, ^1^H magnetic resonance spectroscopy represents a helpful tool for in-vivo tracking of disease-specific pathophysiology.

**Electronic supplementary material:**

The online version of this article (10.1007/s12311-020-01201-y) contains supplementary material, which is available to authorized users.

## Introduction

Spinocerebellar ataxias (SCA) are a group of autosomal-dominant disorders that present as progressive cerebellar ataxia. They have a prevalence of up to 5.6 patients out of 100.000 individuals [1] however with a suspected large number of genetically unconfirmed cases. Depending on the genotype, these disorders lead to atrophy patterns that include the cerebellum and variable regions in the brainstem, basal ganglia, and cerebral cortex. Hence, patients share a cerebellar syndrome but show different concomitant symptoms. The cerebellar involvement and especially the extra-cerebellar degeneration display SCA subtype-specific patterns [2, 3]. The most prevalent SCA subtypes, among them SCA1, are caused by trinucleotide repeat expansion mutations, and the majority presents widespread central nervous system (CNS) involvement [4]. Many among the more recently identified SCA subtypes caused by missense and deletion mutations, such as SCA14, display a cerebellar syndrome with scarce extra-cerebellar manifestations. Neuroimaging studies in SCA14 are confined to case series, and specific atrophy patterns are not well described. SCA14 is caused by missense or deletion mutations in the protein kinase C gamma gene (PRKCG), which is mainly attributed to signal transduction, cell proliferation, and synaptic transmission in Purkinje cells [4]. SCA14 patients suffer from a slowly progressive ataxia. Other symptoms like cognitive impairment, mild sensory impairment, tremor, or axial myoclonus have been reported [5]. SCA1 is caused by a CAG-repeat expansion in the gene ataxin-1 (ATXN1) on chromosome 6p23 [6]. This gene encodes for the protein ataxin-1, which is suggested to regulate gene expression [7]. Besides progressive ataxia, the phenotype includes supranuclear ophthalmoplegia, pyramidal signs, peripheral neuropathy [5], or retinal nerve degeneration [8].

This study was designed based on our previous studies in SCA14 [9] and SCA1 [10]. Compared with these previous total n-acetylaspartate (tNAA) focused studies, we optimized our spectroscopic methods, used a shorter echo time (TE), included more metabolites into the analysis, examined extended patient cohorts of both SCA1 and SCA14, and introduced two cerebellar volumes of interest (VOI) and an extra-cerebellar VOI in the brainstem. In the present study, we aimed to assess metabolic changes in SCA14 and SCA1 patients in both cerebellar and extra-cerebellar regions in order to differentiate them from a control group as well as to identify differences and similarities of these subtypes based on their metabolic profile. An extensive neurochemical profiling might be able to reveal subtype-specific and/or cross-subgroup biomarkers that could be used for facilitation of diagnostic procedures and demonstration of starting points for future therapeutics. Since SCA1 is characterized by widespread neuronal loss and brainstem atrophy [4], we hypothesized that metabolic alterations will be present in the pons. In contrast, we hypothesized that metabolic alterations in SCA14 will be limited to the vermis and cerebellar hemisphere due to the localization of Purkinje cells and expression of protein kinase C gamma. Moreover, we correlated clinical examination scores and characteristics with in vivo levels of each metabolite to check for associations with prognostic significance on the clinical course. For the assessment of brain metabolites, we considered ^1^H magnetic resonance spectroscopy as a powerful tool to analyze the human brain metabolism in vivo. This technique allows for non-invasive detection of biochemical alterations and quantification of metabolites in symptomatic [11] and presymptomatic SCA patients [12]. In addition, ^1^H magnetic resonance spectroscopy is able to distinguish between SCA subtypes [11], and the results of different medical centers are reproducible if identical spectroscopy settings are applied [13].

## Methods

### Participants

We conducted a cross-sectional study with matched healthy controls (HC). Seventeen SCA14 patients with pathogenic variants in PRKCG as well as fourteen SCA1 patients with confirmed pathogenic expansion in ATXN1 were included. Some patients had previously participated in different MRI and ^1^H magnetic resonance spectroscopy studies at our center, published elsewhere [9, 10]. Thirty-one healthy controls were recruited to match each patient to a healthy volunteer of equal gender and similar age (± 3 years). Inclusion criteria for patients were minimum age of 18 years and genetically confirmed diagnosis of SCA14 or SCA1. Exclusion criteria were contraindication for the use of MRI, lack of ability to communicate, and diseases of the CNS other than SCA. Inclusion criterion for healthy controls was minimum age of 18 years, and exclusion criteria were contraindications for the use of MRI as well as neurologic diseases or neurological treatment in the medical background. For all patients, scale for the assessment and rating of ataxia (SARA), score of the cognitive screening test for mild cognitive impairment and early dementia (DemTect), age of onset, and disease duration were obtained. The score of SARA ranges from 0 to 40 with 0 referring to no ataxia and 40 referring to most severe ataxia [14]. If circumstances permitted, SARA was assessed immediately before or after brain scanning. The score of DemTect ranges from 0 to 18 points with 13–18 points corresponding to cognitive power appropriate for age, 9–12 points corresponding to mild cognitive impairment, and 0–8 points corresponding to suspected dementia [15].

### ^1^H Magnetic Resonance Spectroscopy

**Fig. 1:**
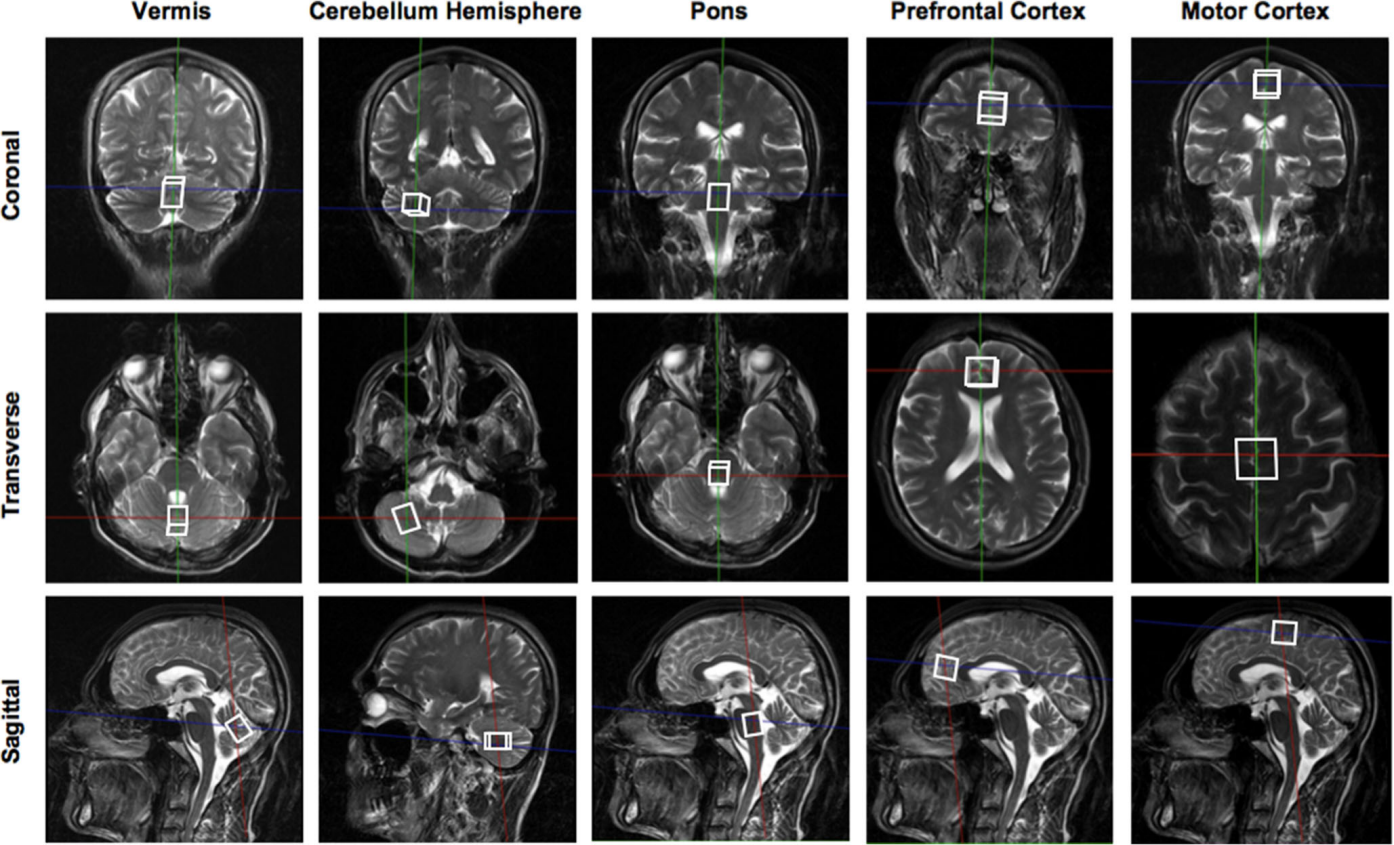
VOI of 3-T single-voxel ^1^H magnetic resonance spectroscopy, which were located in the vermis, right cerebellar hemisphere, pons, prefrontal cortex, and motor cortex. Each VOI is represented in three dimensions coronal, transverse, and sagittal. Size of each VOI was 20 × 20 × 20 mm^3^ in the cerebrocortical regions and 20 × 15 × 15 mm^3^ in the cerebellar locations and pons

Single-voxel ^1^H magnetic resonance spectroscopy was performed using a 3 T scanner (Siemens Magnetom Trio, Erlangen) at the Charité Berlin Center of Advanced Imaging (BCAN). A point resolved spectroscopy sequence (PRESS) was established for stimulation pulses, and the chemical shift selective (CHESS) technique with bandwidth set to 20 Hz was applied in order to suppress the water signal. An TE of 30 ms, which allows assessing metabolites with short relaxation times and a repetition time of 3000 ms were used. In addition to ^1^H magnetic resonance spectroscopy sequences, magnetization-prepared rapid gradient echo (MPRAGE) and sampling perfection with application-optimized contrasts using different flip angle evolution (T2 3D SPACE) sequences were conducted to control for pathologies different from cerebellar atrophy. The standard birdcage one-channel head coil was used for spectroscopy and a 12-channel head coil for MPRAGE and T2 3D SPACE. Sagittal, coronal, and transversal T2-weighted turbo spin-echo MR sequences were used for positioning the VOI. The VOI were positioned in each individual’s brain manually in the vermis, right cerebellar hemisphere, pons, prefrontal cortex, and motor cortex as illustrated in Fig. 1. The VOI in the cerebellar hemisphere included the dentate nucleus, which experiences distinct neuronal loss and atrophy in SCA1 [16, 17] and was positioned right cerebellar in accordance with our previous studies [9, 10]. The VOI localized in extra-cerebellar brain regions were intentionally positioned in the midline in order to maximize the partial volume of cortical brain tissue as well as to have both hemispheres represented within the VOI. We decided against investigating additional VOI in order to not further extend the total scanning time of approximately 1 h. Each VOI measured 20 × 15 × 15 mm^3^ in the cerebellar locations and pons in contrast to 20 × 20 × 20 mm^3^ in the cerebrocortical regions. We intentionally decided not to correct for cerebrospinal fluid in order to avoid additional errors that come along due to error propagation. No morphologically self-evident variations in partial volume effects were seen during planning the VOI.

**Fig. 2:**
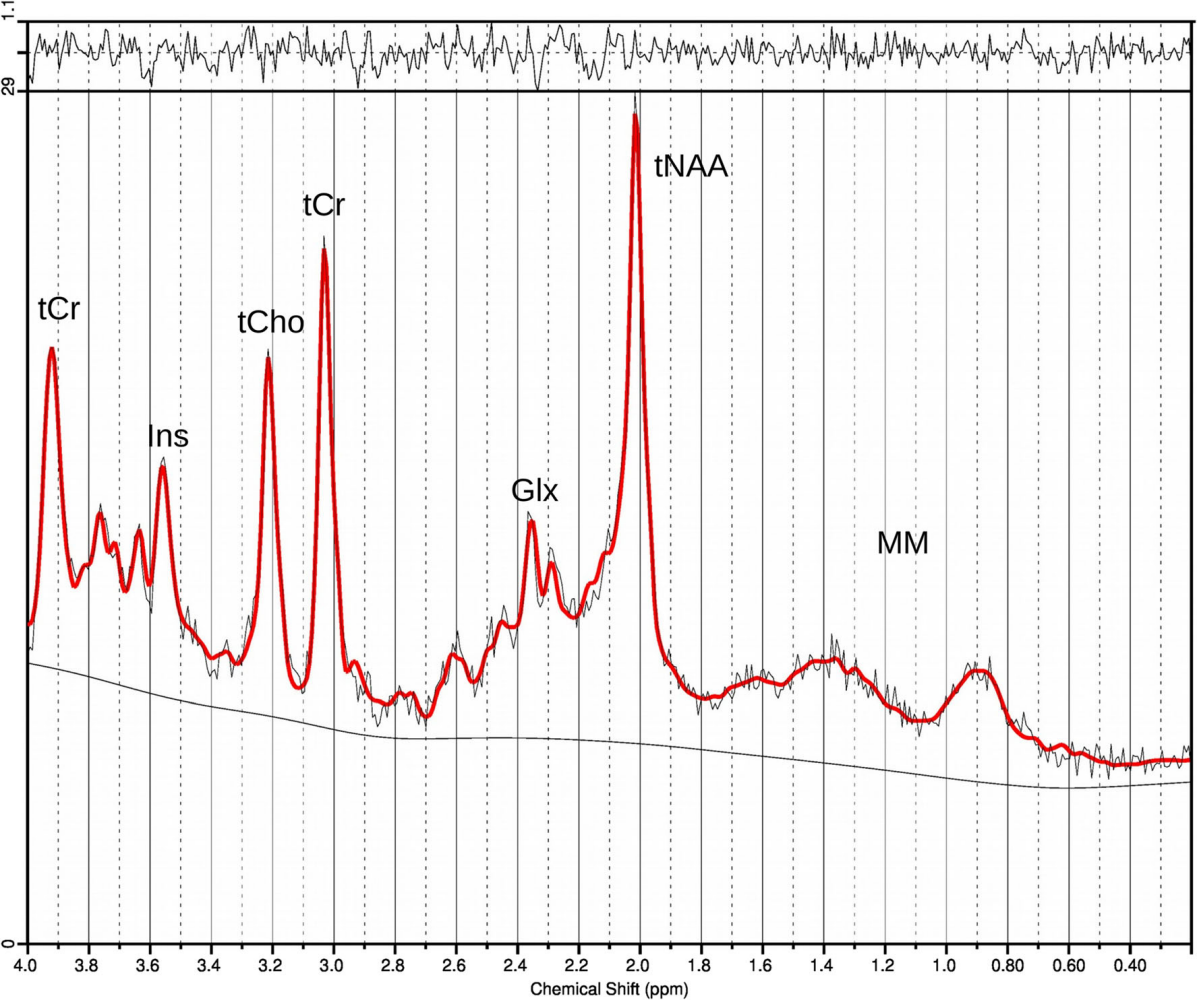
Exemplaric spectrum of the prefrontal cortex from a healthy control included in this study. Total creatine (tCr) results from two spectral peaks (Cr and PCr). Abbreviations: tCr, total creatine; Ins, myo-inositol; tCho, total choline; Glx, glutamate+glutamine; tNAA, total n-acetylaspartate (NAA+NAAG); MM, macromolecules

We quantified the following metabolites: alanine (Ala), aspartate (Asp), creatine (Cr), phosphocreatine (PCr), γ-aminobutyric acid (GABA), glucose (Glc), glutamine (Gln), glutamate (Glu), glycerophosphocholine (GPC), phosphocholine (PCh), glutathione (GSH), myo-inositol (Ins), lactate (Lac), n-acetylaspartate (NAA), n-acetylaspartylglutamate (NAAG), scyllo-inositol (Scyllo), taurine (Tau), negative creatine methylene (CrCH2), total choline (tCho), total n-acetylaspartate (tNAA), total creatine (tCr), and Glu+Gln (Glx). The investigated metabolites are expressed in arbitrary units (AU), because an exact calibration is not achievable. They serve as markers for neuronal density and viability (NAA, NAAG, tNAA), energy metabolism (Glc, Lac, Cr, PCr, tCr), neurotransmission (GABA, Glu, Gln, Glx), glial marker (Ins, Scyllo), membrane turnover and cellularity (GPC, PCh, tCho), antioxidant system (GSH), and amino acids (Ala, Asp, Tau) [18]. CrCH2 represents a correction term. An exemplaric spectrum is given in Fig. 2.

### Statistical Analysis

**Fig. 3:**
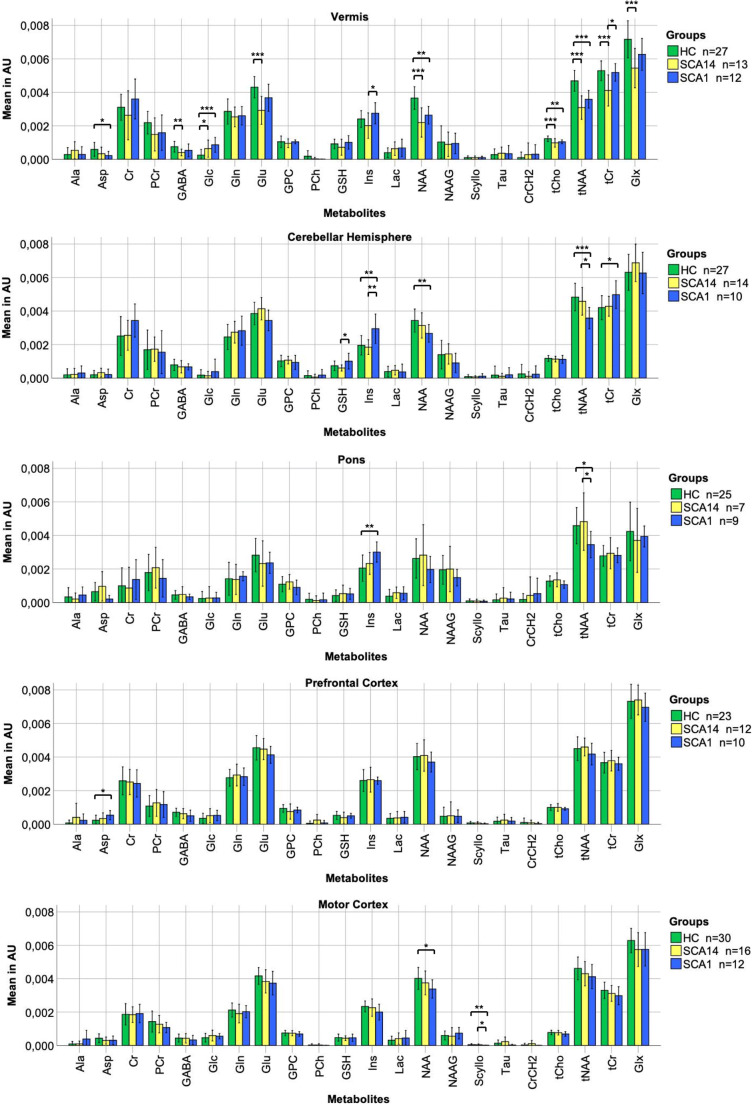
Neurochemical profiles illustrating averaged metabolites of control group, SCA14, and SCA1 for each of the five VOI vermis, cerebellar hemisphere, pons, prefrontal cortex, and motor cortex. Standard deviations are represented as error bars. Significance is marked with * (*p* ≤ 0.05), ** (*p* ≤ 0.01), or *** (*p* ≤ 0.001) asterisks. Abbreviations: HC, healthy controls; AU, arbitrary units; Ala, alanine; Asp, aspartate; Cr, creatine; PCr, phosphocreatine; GABA, γ-aminobutyric acid; Glc, glucose; Gln, glutamine; Glu, glutamate; GPC, glycerophosphorylcholine; PCh, phosphocholine; GSH, glutathione; Ins, myo-inositol; Lac, lactate; NAA, n-acetylaspartate; NAAG, n-acetylaspartylglutamate; Scyllo, scyllo-inositol; Tau, taurine; CrCH2, negative creatine methylene (correction term); tCho, total choline; tNAA, total n-acetylaspartate (NAA+NAAG); tCr, total creatine; Glx, glutamate+glutamine

Quantification of metabolites (Fit) was realized with the LCModel software package (Version 6.3-1L) with a current PRESS basis set for 3 T and TE = 30 ms (delivered by Stephen W. Provencher). As base system, Debian GNU/Linux (version 5.0.10) was used. Mostly, data were not normally distributed as proven by visual impression and Shapiro-Wilk test. As a consequence, Kruskal-Wallis test with adjusted *p* values according to Bonferroni correction was used for group comparisons of metabolites between the three groups of SCA14, SCA1, and HC. Metabolite changes are presented as percentages. For group comparisons of demographic and clinical data, the chi-square test (for female sex), Kruskal-Wallis test (for age at examination), and Mann-Whitney *U* test (for age of onset, disease duration, SARA, and DemTect) were applied. Linear relationships between metabolite levels, and clinical features were evaluated using linear regression analysis with adjustment for age as a major confounding factor. For this analysis, SCA14 and SCA1 cohorts had to be merged due to the small sample size of both patient groups. Statistical analyses and charts were performed using IBM SPSS (www.ibm.com, version 24). A *p* value ≤ 0.05 was considered as significant. Missing data points, but no complete data sets, were excluded from analyses. In order to ensure sufficient measurement accuracy, individual measurements were excluded from the study if spectral bandwidth exceeded 0.125 ppm, signal-to-noise ratio was bad, or significant artifacts were present. Resulting group sizes are given in the text and Fig. 3.

## Results

### Study Population

Groups did not differ in terms of sex, age at examination, age of onset, SARA, and DemTect. However, SCA14 patients revealed a significant longer mean disease duration compared with SCA1 patients (see Table 1). The same was true for the smaller subsamples of HC, SCA14, and SCA1 after elimination of spectra with insufficient quality: SCA14 patients demonstrated significant longer mean disease durations in each VOI compared with SCA1 patients, while the remaining demographic and clinical data were balanced between the groups. The metabolites Glu, Ins, NAA, tCho, tNAA, tCr, and Glx revealed an average Cramer-Rao lower bound (CRLB) of ≤ 20%, allowing for a robust and valid interpretation.

**Table 1:** Demographic data and clinical characteristics of study participants

	HC (*n* = 31)	SCA14 (*n* = 17)	SCA1 (*n* = 14)	*P* value
Female sex, *n* (%)	16 (51.6%)	9 (52.9%)	7 (50.0%)	0.987
Age at examination in years, mean (SD)	48.1 (13.1)	50.9 (12.8)	44.8 (12.4)	0.363
SARA score, mean (SD)		11.0 (3.6)	13.5 (6.9)	0.263
DemTect score, mean (SD)		14.5 (3.6)	12.0 (3.5)	0.264
Age of onset in years, mean (SD)		31.2 (14.8)	36.7 (12.5)	0.325
Disease duration in years, mean (SD)		19.6 (2.9)	7.8 (4.7)	*0.004a*

*HC* healthy controls, *SCA14* spinocerebellar ataxia type 14, *SCA1* spinocerebellar ataxia type 1, *n* quantity, *SD* standard deviation, *SARA* scale for the assessment and rating of ataxia, *DemTect* cognitive screening test for mild cognitive impairment and early dementia, *a* significant group difference in disease duration between SCA14 and SCA1

### Neurochemical Alterations in SCA14

Changes in the neurochemical profile of SCA14 patients revealed no involvement of cerebellar hemisphere or extra-cerebellar structures (pons, prefrontal cortex, or motor cortex). The vermis was the only brain area that showed extensive metabolic changes. Metabolic changes of the vermis (*n* = 13) demonstrated a reduction of tNAA by 34% (*p* < 0.001) and NAA by 40% (*p* < 0.001). Although differentiation between NAA and NAAG is difficult using ^1^H magnetic resonance spectroscopy, we detected no changes in NAAG. Besides, we report about markers associated with energy metabolism alterations. SCA14 patients showed a reduction in tCr by 22% (*p* = 0.001), Glu by 32% (*p* < 0.001), Glx by 24% (*p* < 0.001), and GABA by 46% (*p* = 0.003) in the vermis. There was no change of Gln in the vermis. However, differentiation of Glu and Gln is difficult, and reliability is limited using 3 T ^1^H magnetic resonance spectroscopy. Consistent with energy metabolism changes, we identified an elevation of Glc by 155% (*p* = 0.026) in the vermis. Furthermore, tCho was reduced by 20% (*p* = 0.001) in the vermis. In contrast to SCA1 spectra, no significant alterations were identified for Asp and Ins in SCA14.

### Neurochemical Alterations in SCA1

In SCA1, we identified extended metabolic changes detectable in each VOI with a focus on vermis, cerebellar hemisphere, and pons. We analyzed *n* = 12 patients in the vermis, *n* = 10 in the cerebellar hemisphere, *n* = 9 in the pons, *n* = 10 in the prefrontal cortex, and *n* = 12 in the motor cortex after exclusion of spectra with insufficient quality. Here, we detected a tNAA reduction by 24% in the vermis (*p* < 0.001), by 26% in the cerebellar hemisphere (*p* = 0.001), and by 25% in the pons (*p* = 0.017), but not in the prefrontal or motor cortices. NAA was decreased by 28% in vermis (*p* = 0.002), by 22% in the cerebellar hemisphere (*p* = 0.007), and by 16% in the motor cortex (*p* = 0.015). In addition, our SCA1 patient cohort revealed an elevation of Ins by 51% in the cerebellar hemisphere (*p* = 0.007) and by 46% in the pons (*p* = 0.005). No changes in Ins were detectable in the vermis. The energy metabolism showed an elevation of tCr by 18% in the cerebellar hemisphere (*p* = 0.028) as well as an elevation of Glc by 247% in the vermis (*p* < 0.001). Moreover, the vermis of SCA1 patients presented with a reduction of tCho by 15% (p = 0.007) and a tendency for Glu reduction by 15% (*p* = 0.086). Besides, we identified an Asp reduction by 62% in the vermis (*p* = 0.019) and a marked elevation by 130% in the prefrontal cortex (*p* = 0.025). In the motor cortex, Scyllo was reduced by 90% in SCA1 patients (*p* = 0.003). In contrast to SCA14 spectra, none of the investigated brain areas revealed significant changes in GABA or Glx levels in SCA1.

### Neurochemical Differences Between SCA14 and SCA1

In the vermis, SCA14 patients revealed 21% lower tCr in comparison with SCA1 patients (*p* = 0.025). Compared with SCA14, SCA1 patients showed an elevation of Ins by 37% in the vermis (*p* = 0.015) and by 59% in the cerebellar hemisphere (*p* = 0.007). In the cerebellar hemisphere, 67% higher GSH (*p* = 0.038) and a trend for 17% lower Glu (*p* = 0.051) were detectable in SCA1 compared with SCA14. In addition, SCA1 patients demonstrated a tNAA reduction by 22% in the cerebellar hemisphere (*p* = 0.046) and by 28% in the pons (*p* = 0.014) in relation to SCA14 patients. There were no metabolic differences in the prefrontal cortex. In the motor cortex, SCA1 patients presented with 88% lower Scyllo (*p* = 0.039) and a tendency for 90% lower Tau (*p* = 0.055) compared with SCA14 patients.

### Regression Analysis with Adjustment for Age

#### SARA

In the cerebellar hemisphere and pons, bad motor performance (high SARA scores) were linked to low NAA (*p* = 0.048; *p* = 0.055), tNAA (*p* = 0.011, *p* = 0.028), and tCho (*p* = 0.007; *p* = 0.015). Also, high SARA scores were associated with high Asp level in the prefrontal cortex (*p* = 0.005).

#### DemTect

Better cognition (higher DemTect score) correlated significant with higher levels of NAAG (*p* = 0.016) and tNAA (*p* = 0.006) in the cerebellar hemisphere as well as correlated weakly with higher levels of NAA (*p* = 0.064) and tNAA (*p* = 0.063) in the pons. Moreover, higher DemTect score was associated with higher tCho in the pons (*p* = 0.047) and a tendency for higher tCho in the motor cortex (*p* = 0.081). In addition, higher Gln (*p* = 0.024) and a tendency for higher Glx (*p* = 0.086) in the motor cortex were linked to better cognitive power (higher DemTect score). Last, there was a weak association of better cognition (high DemTect score) with lower Glc level in the prefrontal cortex (*p* = 0.073).

#### Age of Onset and Disease Duration

High Ins and high tCho in the cerebellar hemisphere were weakly associated with higher age of onset (*p* = 0.050; *p* = 0.054) and shorter disease duration (*p* = 0.055; *p* = 0.055). In contrast, patients with high Ins and high tCho in the motor cortex showed tendencies for lower age at onset (*p* = 0.071; *p* = 0.067) and longer disease duration (*p* = 0.071; *p* = 0.068). Also, high NAA and low NAAG in the motor cortex were linked to lower age of onset (*p* = 0.016; *p* = 0.012) and longer disease duration (*p* = 0.016; *p* = 0.012). No association was seen for tNAA here. In the pons, higher Glc correlated with lower age at onset (*p* = 0.042) and longer disease duration (*p* = 0.042).

## Discussion

This study assessed metabolic changes in cerebellar and extra-cerebellar brain regions in SCA1 4 and SCA1 patients using 3-T single-voxel ^1^H magnetic resonance spectroscopy. In-vivo metabolites were compared between the three groups of SCA14, SCA1, and HC as well as linear relationships between metabolite levels, and clinical features were evaluated for merged patient groups with adjustment for age.

In SCA14, metabolic changes were strongly restricted to the vermis, while extra-cerebellar brain regions and particularly the cerebellar hemisphere were not involved in neurochemical alterations. In the vermis of SCA14 patients, tNAA, tCho, tCr, GABA, and Glu were mainly decreased as well as Glc was increased. The tCho reduction and Glc elevation have not been described for SCA14 yet, while the reductions in tNAA, tCr, GABA, and Glu confirm previous research [9]. The tNAA reduction is characteristically for neurodegenerative diseases and corresponds to neuronal cell loss or neuronal dysfunction [18]. Low tCr may indicate suppressed energy metabolism with a consecutive accumulation of Glc and low membrane turnover as illustrated by tCho reduction. The decreased neurotransmitters GABA and Glu may reflect loss of GABAergic Purkinje cells and loss of climbing fibers, whose efferences maintain glutamatergic synapses with Purkinje cells.

SCA1 patients demonstrated similar results in vermis: There was a significant reduction in tNAA and tCho but less pronounced than in SCA14. Although results on tCho levels in SCA1 are heterogeneous, the observed tCho reduction of the present study supports previous indications for low tCho in the cerebellum [19, 20]. Alike SCA14, Glu was reduced in the vermis, but did not reach statistical significance in SCA1 likely due to significant longer disease duration of the SCA14 cohort. Furthermore, both subtypes had a Glc elevation in common, which was more pronounced in the SCA1 than in the SCA14 cohort (247% vs. 155%). Supporting these results, Glc levels were elevated in the cerebellum of mice overexpressing mutant human ataxin-1 [21]. Also, SCA1 patients revealed an increase of Glc+tau amounts in the vermis [12] and cerebellar hemisphere [12, 22]. In positron emission tomography, a reduced Glc metabolism was seen in the cerebellum and brainstem of SCA1 patients [23]. In fact, a Glc hypometabolism has also been reported for other polyglutamine diseases such as SCA2, SCA3, SCA6 [24], and Huntington’s disease [25] as well as for neurodegenerative diseases without trinucleotide repeats such as Alzheimer’s disease [26], amyotrophic lateral sclerosis [27], and Parkinson’s disease [28]. Accordingly, the Glc elevation in the vermis could represent an accumulation due to a reduced Glc metabolism. In distinction from SCA14, Asp was reduced, and tCr was not changed in the vermis of SCA1 patients. Interestingly, aspartate-glutamate homeostasis in neuronal cells is affected by the availability of glucose and ketone bodies. In the presence of ketone bodies only, neurons showed higher aspartate and lower glutamate levels compared with presence of glucose alone [29]. Thus, high levels of Glc may affect aspartate levels in the vermis. However, Asp also serves as substrate for NAA synthesis, and low Asp could simply be linked to low NAA levels [30]. Since the SCA1 cohort was characterized by distinct changes in Glc and Asp as opposed to SCA14, despite a significant shorter disease duration, these metabolites might point to metabolic pathways differing between SCA14 and SCA1.

In SCA1 patients, more widespread metabolic changes were present. The tNAA reduction was not only found in vermis of SCA1 patients but also in the cerebellar hemisphere, pons, and as a trend in the motor cortex. Furthermore, the SCA1 cohort featured an Ins elevation in the cerebellar hemisphere and pons, whereas the vermis was excluded from an increase. This result confirms previous studies [12, 22]. Ins is considered a marker for glial proliferation or hyperplasia in the sense of gliosis [18]. High Ins levels could indicate an involvement of glial cells in the pathomechanism of SCA1. In fact, the Bergmann glia of a SCA1 mouse model revealed a reduced expression of the Glu-Asp transporter (GLAST) that correlated with the loss of Purkinje neurons [31]. Moreover, Asp showed a reduction not only in the vermis of SCA1 patients but also in the pons, which did not reach statistical significance probably due to the small sample size in pons (*n* = 9). The pons is anatomically located in an area, where shimming in the majority of cases does not result in sufficient B_0_ field homogeneity and subsequently only a small number of data sets met sufficient spectral quality. Interestingly, each metabolite of tNAA, Ins, and Asp was altered to the same extent across the affected brain areas in the metencephalon (tNAA in the vermis − 24%, cerebellar hemisphere − 26%, and pons − 25%; Ins in the cerebellar hemisphere + 51% and pons + 46%; Asp in the vermis − 62% and pons − 68%). However, no changes were identified for Glu, Glc, and tCho in SCA1 outside the vermis. In contrast to the reduction of tCr in the vermis of SCA14 patients, but in line with previous research [22], we found a tCr elevation selectively in the cerebellar hemisphere of SCA1 patients. Adanyeguh and colleagues reported about tCr increases in the vermis and pons of SCA1 without investigating the cerebellar hemisphere [32]. Since tCr levels are related to mental activity [33], the discrepancy in the distribution of tCr elevations might be attributed to recently varying activity states. TCr is considered a marker for energy metabolism and serves as buffer for adenosine triphosphate (ATP) [30]. Besides a Glc hypometabolism in the cerebellum of SCA1 patients, downregulation of certain enzymes involved in glycolysis and ATP synthesis, lower ATP levels [34], and reduced activities of the respiratory chain complexes [35] were identified in the cerebellum of SCA1 mouse models. Therefore, high tCr could be a reaction on suppressed energy metabolism in order to maintain fast energy supply in case of high energy demand. Moreover, Scyllo was reduced in the motor cortex of SCA1 patients, which should be reevaluated in future studies.

Neurochemical changes were observed in SCA14 selectively in the vermis and in SCA1 throughout the cerebellum and pons. Besides changes related to the different atrophy patterns, both subtypes differed by direct comparison in the metabolites tCr, Ins, and GSH in the cerebellum as well as Scyllo and Tau in the motor cortex. Since GSH and Tau were unchanged in both patient groups compared with the control group, these results seem questionable.

Regression analysis did not reveal any significant associations between clinical data and metabolite levels in the vermis. This could be explained by focal atrophy with distinct loss of cerebellar tissue and consequently very low metabolite levels. Of note, the provided analysis tested only for linear relationships. In the cerebellar hemisphere as well as in the pons, low tNAA levels were associated with worse cognitive power (lower DemTect scores) and, in accordance with a previous study [22], worse motor performances (higher SARA scores). Although no alterations in tCho were found outside the vermis, low tCho in the cerebellar hemisphere and/or pons were indicative for poorer clinical presentation (worse SARA, worse DemTect, lower age of onset, longer disease duration). Moreover, weak associations between high Ins level in the cerebellar hemisphere and higher age of onset, respectively, shorter disease duration were identified. Since SCA14 did not reveal alterations in Ins, these associations seem to be an effect of the SCA1 cohort. Glc showed only isolated associations with clinical parameters, which were merely present in brain areas without altered Glc levels. Further investigations on Ins, tCho, and Glc levels in SCA1 and SCA14 patients are needed to confirm these results. With regard to Deelchand and colleagues [13], a multi-center study could overcome the issue of underpowered patient cohorts and clearly enhance the quality of analysis. Future studies using ^1^H magnetic resonance spectroscopy would also benefit from a two-staged data collection for evaluation of metabolite fluctuations/courses over time, a combination of spectroscopic with structural MRI measurements, and an inclusion of presymptomatic mutation carriers in addition to symptomatic patients.

The various SCA genotypes share cerebellar ataxia as a joint clinical characteristic and cerebellar atrophy of yet different degree and pattern. Beyond, SCA patients may share disease features common with other neurodegenerative diseases, especially mitochondrial disorders. Against this background, it seems possible that different SCA genotypes share pathological mechanisms and pathways. It remains to be shown, if SCAs with similar atrophy patterns share more pathophysiological pathways than SCAs with different patterns. Our study examined two SCA subtypes with different atrophy patterns. Still, we found common metabolic changes that are in line with previous results and might be attributed to dysfunctional energy metabolism in both SCA14 and SCA1. Since mitochondrial and glycolytic pathways have great impact on neuronal energy metabolism, these findings offer an eligible approach for further investigations in SCA14 and SCA1.

## Electronic Supplementary Material

ESM 1 (DOC 504 kb).

## Data Availability

The data set analyzed in this study is available from the corresponding author on a reasonable request.
