# Supplementary material for: Neurochemical Differences in Spinocerebellar Ataxia Type 14 and 1
Source: Cerebellum. 2020 Oct 15;20(2):169–78. doi: 10.1007/s12311-020-01201-y (PMC8004522; doi:10.1007/s12311-020-01201-y)
Supplement: Supplementary file 1 — (DOC 504 kb). [file 12311_2020_1201_MOESM1_ESM.doc]

**Supplementary material**

**Table S1** Comparisons of metabolite levels between the three groups of healthy controls (HC), SCA14, and SCA1. Means and standard deviations (SD) in arbitrary units (AU) as well as p-values are displayed for each VOI and metabolite.

**Table S1a** Vermis.

| **Metabolites** | | **HC** | **SCA14** | **SCA1** | **P-value**  **SCA14-HC** | **P-value**  **SCA1-HC** | **P-value**  **SCA14-SCA1** |
| --- | --- | --- | --- | --- | --- | --- | --- |
| Ala | Mean | 28.3 · 10^-5^ | 53.3 · 10^-5^ | 28.7 · 10^-5^ | 0.628 | 1.000 | 0.754 |
|  | SD | 41.2 · 10^-5^ | 50.9 · 10^-5^ | 45.5 · 10^-5^ |  |  |  |
| Asp | Mean | 58.7 · 10^-5^ | 33.1 · 10^-5^ | 22.1 · 10^-5^ | 0.138 | **0.019** | 1.000 |
|  | SD | 41.9 · 10^-5^ | 39.3 · 10^-5^ | 24.3 · 10^-5^ |  |  |  |
| Cr | Mean | 310.7 · 10^-5^ | 262.6 · 10^-5^ | 360.3 · 10^-5^ | 0.546 | 0.894 | 0.128 |
|  | SD | 77.9 · 10^-5^ | 147.0 · 10^-5^ | 119.3 · 10^-5^ |  |  |  |
| PCr | Mean | 218.5 · 10^-5^ | 148.2 · 10^-5^ | 158.6 · 10^-5^ | 0.069 | 0.175 | 1.000 |
|  | SD | 67.2 · 10^-5^ | 98.8 · 10^-5^ | 106.0 · 10^-5^ |  |  |  |
| GABA | Mean | 74.9 · 10^-5^ | 40.5 · 10^-5^ | 53.0 · 10^-5^ | **0.003** | 0.233 | 0.662 |
|  | SD | 31.9 · 10^-5^ | 19.8 · 10^-5^ | 38.1 · 10^-5^ |  |  |  |
| Glc | Mean | 24.9 · 10^-5^ | 63.5 · 10^-5^ | 86.5 · 10^-5^ | **0.026** | **<0.001** | 0.802 |
|  | SD | 33.4 · 10^-5^ | 51.4 · 10^-5^ | 44.6 · 10^-5^ |  |  |  |
| Gln | Mean | 285.8 · 10^-5^ | 253.2 · 10^-5^ | 259.8 · 10^-5^ | 0.713 | 1.000 | 1.000 |
|  | SD | 75.0 · 10^-5^ | 58.9 · 10^-5^ | 55.3 · 10^-5^ |  |  |  |
| Glu | Mean | 430.8 · 10^-5^ | 292.0 · 10^-5^ | 367.4 · 10^-5^ | **<0.001** | 0.086 | 0.246 |
|  | SD | 63.0 · 10^-5^ | 83.2 · 10^-5^ | 81.2 · 10^-5^ |  |  |  |
| GPC | Mean | 104.5 · 10^-5^ | 95.4 · 10^-5^ | 103.9 · 10^-5^ | 0.264 | 1.000 | 1.000 |
|  | SD | 34.5 · 10^-5^ | 26.0 · 10^-5^ | 11.6 · 10^-5^ |  |  |  |
| PCh | Mean | 18.2 · 10^-5^ | 2.4 · 10^-5^ | <0.1 · 10^-5^ | 0.236 | 0.075 | 1.000 |
|  | SD | 32.9 · 10^-5^ | 8.8 · 10^-5^ | <0.1 · 10^-5^ |  |  |  |
| GSH | Mean | 91.5 · 10^-5^ | 71.5 · 10^-5^ | 100.6 · 10^-5^ | 0.156 | 1.000 | 0.146 |
|  | SD | 28.0 · 10^-5^ | 47.9 · 10^-5^ | 40.4 · 10^-5^ |  |  |  |
| Ins | Mean | 240.5 · 10^-5^ | 201.0 · 10^-5^ | 275.2 · 10^-5^ | 0.198 | 0.442 | **0.015** |
|  | SD | 50.0 · 10^-5^ | 76.1 · 10^-5^ | 63.7 · 10^-5^ |  |  |  |
| Lac | Mean | 39.3 · 10^-5^ | 62.9 · 10^-5^ | 67.7 · 10^-5^ | 0.277 | 0.197 | 1.000 |
|  | SD | 28.6 · 10^-5^ | 40.6 · 10^-5^ | 52.0 · 10^-5^ |  |  |  |
| NAA | Mean | 365.8 · 10^-5^ | 219.5 · 10^-5^ | 263.4 · 10^-5^ | **<0.001** | **0.002** | 1.000 |
|  | SD | 67.5 · 10^-5^ | 87.6 · 10^-5^ | 52.6 · 10^-5^ |  |  |  |
| NAAG | Mean | 102.9 · 10^-5^ | 89.3 · 10^-5^ | 94.5 · 10^-5^ | 1.000 | 1.000 | 1.000 |
|  | SD | 97.0 · 10^-5^ | 73.3 · 10^-5^ | 61.1 · 10^-5^ |  |  |  |

| Scyllo | Mean | 9.9 · 10^-5^ | 9.3 · 10^-5^ | 9.7 · 10^-5^ | 1.000 | 1.000 | 1.000 |
| --- | --- | --- | --- | --- | --- | --- | --- |
|  | SD | 10.6 · 10^-5^ | 10.3 · 10^-5^ | 8.5 · 10^-5^ |  |  |  |
| Tau | Mean | 27.9 · 10^-5^ | 35.8 · 10^-5^ | 33.3 · 10^-5^ | 1.000 | 1.000 | 1.000 |
|  | SD | 33.7 · 10^-5^ | 36.9 · 10^-5^ | 48.8 · 10^-5^ |  |  |  |
| CrCH2 | Mean | 10.0 · 10^-5^ | 27.4 · 10^-5^ | 30.1 · 10^-5^ | 0.758 | 1.000 | 1.000 |
|  | SD | 33.3 · 10^-5^ | 69.6 · 10^-5^ | 56.7 · 10^-5^ |  |  |  |
| tCho | Mean | 122.7 · 10^-5^ | 97.8 · 10^-5^ | 103.9 · 10^-5^ | **0.001** | **0.007** | 1.000 |
|  | SD | 16.3 · 10^-5^ | 25.4 · 10^-5^ | 11.6 · 10^-5^ |  |  |  |
| tNAA | Mean | 468.7 · 10^-5^ | 308.9 · 10^-5^ | 358.3 · 10^-5^ | **<0.001** | **<0.001** | 1.000 |
|  | SD | 62.2 · 10^-5^ | 70.3 · 10^-5^ | 52.9 · 10^-5^ |  |  |  |
| tCr | Mean | 529.3 · 10^-5^ | 410.9 · 10^-5^ | 518.9 · 10^-5^ | **0.001** | 1.000 | **0.025** |
|  | SD | 58.6 · 10^-5^ | 93.0 · 10^-5^ | 52.3 · 10^-5^ |  |  |  |
| Glx | Mean | 716.6 · 10^-5^ | 545.0 · 10^-5^ | 627.0 · 10^-5^ | **<0.001** | 0.114 | 0.413 |
|  | SD | 111.2 · 10^-5^ | 118.0 · 10^-5^ | 95.2 · 10^-5^ |  |  |  |

**Table S1b** Cerebellar hemisphere.

| **Metabolites** | | **HC** | **SCA14** | **SCA1** | **P-value**  **SCA14-HC** | **P-value**  **SCA1-HC** | **P-value**  **SCA14-SCA1** |
| --- | --- | --- | --- | --- | --- | --- | --- |
| Ala | Mean | 19.7 · 10^-5^ | 22.0 · 10^-5^ | 30.2 · 10^-5^ | 1.000 | 0.735 | 1.000 |
|  | SD | 36.2 · 10^-5^ | 35.7 · 10^-5^ | 42.6 · 10^-5^ |  |  |  |
| Asp | Mean | 19.8 · 10^-5^ | 32.4 · 10^-5^ | 21.4 · 10^-5^ | 0.214 | 1.000 | 0.403 |
|  | SD | 25.4 · 10^-5^ | 26.3 · 10^-5^ | 32.6 · 10^-5^ |  |  |  |
| Cr | Mean | 251.2 · 10^-5^ | 255.5 · 10^-5^ | 343.6 · 10^-5^ | 1.000 | 0.095 | 0.260 |
|  | SD | 116.1 · 10^-5^ | 89.5 · 10^-5^ | 98.9 · 10^-5^ |  |  |  |
| PCr | Mean | 169.2 · 10^-5^ | 172.8 · 10^-5^ | 154.6 · 10^-5^ | 1.000 | 1.000 | 1.000 |
|  | SD | 117.8 · 10^-5^ | 72.4 · 10^-5^ | 128.3 · 10^-5^ |  |  |  |
| GABA | Mean | 78.8 · 10^-5^ | 67.2 · 10^-5^ | 67.2 · 10^-5^ | 0.915 | 0.781 | 1.000 |
|  | SD | 33.8 · 10^-5^ | 36.8 · 10^-5^ | 18.3 · 10^-5^ |  |  |  |
| Glc | Mean | 18.3 · 10^-5^ | 14.0 · 10^-5^ | 38.9 · 10^-5^ | 1.000 | 1.000 | 1.000 |
|  | SD | 32.5 · 10^-5^ | 26.2 · 10^-5^ | 74.6 · 10^-5^ |  |  |  |
| Gln | Mean | 245.7 · 10^-5^ | 274.0 · 10^-5^ | 282.8 · 10^-5^ | 0.820 | 0.783 | 1.000 |
|  | SD | 74.3 · 10^-5^ | 65.3 · 10^-5^ | 86.7 · 10^-5^ |  |  |  |
| Glu | Mean | 385.5 · 10^-5^ | 413.9 · 10^-5^ | 344.2 · 10^-5^ | 0.541 | 0.416 | 0.051 |
|  | SD | 66.9 · 10^-5^ | 66.5 · 10^-5^ | 61.9 · 10^-5^ |  |  |  |
| GPC | Mean | 102.5 · 10^-5^ | 107.2 · 10^-5^ | 93.6 · 10^-5^ | 1.000 | 1.000 | 1.000 |
|  | SD | 33.9 · 10^-5^ | 22.7 · 10^-5^ | 42.4 · 10^-5^ |  |  |  |
| PCh | Mean | 15.2 · 10^-5^ | 6.3 · 10^-5^ | 18.5 · 10^-5^ | 1.000 | 1.000 | 0.963 |
|  | SD | 28.4 · 10^-5^ | 16.8 · 10^-5^ | 32.8 · 10^-5^ |  |  |  |
| GSH | Mean | 73.5 · 10^-5^ | 60.4 · 10^-5^ | 101.1 · 10^-5^ | 0.778 | 0.224 | **0.038** |
|  | SD | 28.7 · 10^-5^ | 18.5 · 10^-5^ | 46.9 · 10^-5^ |  |  |  |
| Ins | Mean | 195.3 · 10^-5^ | 184.9 · 10^-5^ | 294.7 · 10^-5^ | 1.000 | **0.007** | **0.007** |
|  | SD | 58.1 · 10^-5^ | 44.0 · 10^-5^ | 87.5 · 10^-5^ |  |  |  |
| Lac | Mean | 38.7 · 10^-5^ | 46.6 · 10^-5^ | 36.9 · 10^-5^ | 1.000 | 1.000 | 0.697 |
|  | SD | 31.5 · 10^-5^ | 27.9 · 10^-5^ | 46.7 · 10^-5^ |  |  |  |
| NAA | Mean | 343.7 · 10^-5^ | 314.2 · 10^-5^ | 266.9 · 10^-5^ | 0.909 | **0.007** | 0.178 |
|  | SD | 68.9 · 10^-5^ | 75.6 · 10^-5^ | 52.6 · 10^-5^ |  |  |  |
| NAAG | Mean | 139.6 · 10^-5^ | 144.3 · 10^-5^ | 90.8 · 10^-5^ | 1.000 | 0.265 | 0.231 |
|  | SD | 85.1 · 10^-5^ | 60.6 · 10^-5^ | 57.2 · 10^-5^ |  |  |  |
| Scyllo | Mean | 8.9 · 10^-5^ | 6.9 · 10^-5^ | 11.2 · 10^-5^ | 1.000 | 1.000 | 1.000 |
|  | SD | 11.6 · 10^-5^ | 7.2 · 10^-5^ | 15.7 · 10^-5^ |  |  |  |
| Tau | Mean | 18.2 · 10^-5^ | 10.5 · 10^-5^ | 19.8 · 10^-5^ | 1.000 | 1.000 | 1.000 |
|  | SD | 53.4 · 10^-5^ | 18.2 · 10^-5^ | 42.6 · 10^-5^ |  |  |  |
| CrCH2 | Mean | 25.3 · 10^-5^ | 10.7 · 10^-5^ | 22.9 · 10^-5^ | 1.000 | 1.000 | 1.000 |
|  | SD | 56.0 · 10^-5^ | 27.4 · 10^-5^ | 50.2 · 10^-5^ |  |  |  |
| tCho | Mean | 117.7 · 10^-5^ | 113.5 · 10^-5^ | 112.1 · 10^-5^ | 1.000 | 1.000 | 1.000 |
|  | SD | 17.2 · 10^-5^ | 16.8 · 10^-5^ | 23.6 · 10^-5^ |  |  |  |
| tNAA | Mean | 483.5 · 10^-5^ | 458.3 · 10^-5^ | 358.0 · 10^-5^ | 0.983 | **0.001** | **0.046** |
|  | SD | 82.3 · 10^-5^ | 83.0 · 10^-5^ | 64.2 · 10^-5^ |  |  |  |
| tCr | Mean | 420.5 · 10^-5^ | 428.4 · 10^-5^ | 498.0 · 10^-5^ | 1.000 | **0.028** | 0.147 |
|  | SD | 71.7 · 10^-5^ | 59.3 · 10^-5^ | 82.5 · 10^-5^ |  |  |  |
| Glx | Mean | 631.2 · 10^-5^ | 687.8 · 10^-5^ | 626.9 · 10^-5^ | 0.238 | 1.000 | 0.318 |
|  | SD | 107.1 · 10^-5^ | 111.9 · 10^-5^ | 123.4 · 10^-5^ |  |  |  |

**Table S1c** Pons.

| **Metabolites** | | **HC** | **SCA14** | **SCA1** | **P-value**  **SCA14-HC** | **P-value**  **SCA1-HC** | **P-value**  **SCA14-SCA1** |
| --- | --- | --- | --- | --- | --- | --- | --- |
| Ala | Mean | 33.3 · 10^-5^ | 20.8 · 10^-5^ | 44.6 · 10^-5^ | 1.000 | 0.953 | 0.578 |
|  | SD | 55.5 · 10^-5^ | 36.1 · 10^-5^ | 48.1 · 10^-5^ |  |  |  |
| Asp | Mean | 64.7 · 10^-5^ | 96.6 · 10^-5^ | 20.6 · 10^-5^ | 1.000 | 0.129 | 0.101 |
|  | SD | 54.7 · 10^-5^ | 88.0 · 10^-5^ | 21.2 · 10^-5^ |  |  |  |
| Cr | Mean | 99.7 · 10^-5^ | 86.3 · 10^-5^ | 137.3 · 10^-5^ | 1.000 | 0.920 | 0.828 |
|  | SD | 107.5 · 10^-5^ | 124.3 · 10^-5^ | 118.5 · 10^-5^ |  |  |  |
| PCr | Mean | 178.8 · 10^-5^ | 207.8 · 10^-5^ | 144.0 · 10^-5^ | 1.000 | 1.000 | 0.651 |
|  | SD | 108.8 · 10^-5^ | 121.6 · 10^-5^ | 111.8 · 10^-5^ |  |  |  |
| GABA | Mean | 45.4 · 10^-5^ | 48.0 · 10^-5^ | 34.1 · 10^-5^ | 1.000 | 0.752 | 1.000 |
|  | SD | 25.8 · 10^-5^ | 46.6 · 10^-5^ | 15.6 · 10^-5^ |  |  |  |
| Glc | Mean | 24.1 · 10^-5^ | 26.1 · 10^-5^ | 26.9 · 10^-5^ | 0.748 | 1.000 | 0.314 |
|  | SD | 42.2 · 10^-5^ | 69.2 · 10^-5^ | 34.0 · 10^-5^ |  |  |  |
| Gln | Mean | 141.9 · 10^-5^ | 137.5 · 10^-5^ | 157.0 · 10^-5^ | 1.000 | 1.000 | 1.000 |
|  | SD | 98.0 · 10^-5^ | 90.0 · 10^-5^ | 26.9 · 10^-5^ |  |  |  |
| Glu | Mean | 282.2 · 10^-5^ | 232.7 · 10^-5^ | 236.9 · 10^-5^ | 1.000 | 0.389 | 1.000 |
|  | SD | 99.5 · 10^-5^ | 135.1 · 10^-5^ | 63.3 · 10^-5^ |  |  |  |
| GPC | Mean | 109.1 · 10^-5^ | 123.0 · 10^-5^ | 90.2 · 10^-5^ | 1.000 | 0.754 | 0.272 |
|  | SD | 45.4 · 10^-5^ | 43.4 · 10^-5^ | 43.8 · 10^-5^ |  |  |  |
| PCh | Mean | 19.0 · 10^-5^ | 11.9 · 10^-5^ | 16.8 · 10^-5^ | 1.000 | 1.000 | 1.000 |
|  | SD | 36.1 · 10^-5^ | 28.3 · 10^-5^ | 39.5 · 10^-5^ |  |  |  |
| GSH | Mean | 41.5 · 10^-5^ | 53.5 · 10^-5^ | 51.6 · 10^-5^ | 1.000 | 1.000 | 1.000 |
|  | SD | 34.5 · 10^-5^ | 50.4 · 10^-5^ | 32.9 · 10^-5^ |  |  |  |
| Ins | Mean | 205.5 · 10^-5^ | 233.0 · 10^-5^ | 301.0 · 10^-5^ | 1.000 | **0.005** | 0.263 |
|  | SD | 78.0 · 10^-5^ | 64.9 · 10^-5^ | 60.4 · 10^-5^ |  |  |  |
| Lac | Mean | 38.1 · 10^-5^ | 58.6 · 10^-5^ | 55.4 · 10^-5^ | 0.454 | 0.417 | 1.000 |
|  | SD | 39.9 · 10^-5^ | 33.8 · 10^-5^ | 38.7 · 10^-5^ |  |  |  |
| NAA | Mean | 262.9 · 10^-5^ | 282.8 · 10^-5^ | 197.1 · 10^-5^ | 1.000 | 0.251 | 0.211 |
|  | SD | 117.4 · 10^-5^ | 181.7 · 10^-5^ | 78.2 · 10^-5^ |  |  |  |
| NAAG | Mean | 195.3 · 10^-5^ | 199.4 · 10^-5^ | 148.6 · 10^-5^ | 1.000 | 0.366 | 0.845 |
|  | SD | 86.0 · 10^-5^ | 135.4 · 10^-5^ | 49.8 · 10^-5^ |  |  |  |
| Scyllo | Mean | 10.0 · 10^-5^ | 9.0 · 10^-5^ | 7.5 · 10^-5^ | 1.000 | 1.000 | 1.000 |
|  | SD | 11.5 · 10^-5^ | 10.9 · 10^-5^ | 7.2 · 10^-5^ |  |  |  |
| Tau | Mean | 16.3 · 10^-5^ | 26.1 · 10^-5^ | 21.5 · 10^-5^ | 1.000 | 1.000 | 1.000 |
|  | SD | 34.8 · 10^-5^ | 62.8 · 10^-5^ | 39.9 · 10^-5^ |  |  |  |
| CrCH2 | Mean | 18.5 · 10^-5^ | 41.9 · 10^-5^ | 53.5 · 10^-5^ | 1.000 | 1.000 | 0.758 |
|  | SD | 35.2 · 10^-5^ | 110.7 · 10^-5^ | 91.7 · 10^-5^ |  |  |  |
| tCho | Mean | 128.1 · 10^-5^ | 134.9 · 10^-5^ | 107.0 · 10^-5^ | 1.000 | 0.189 | 0.094 |
|  | SD | 30.7 · 10^-5^ | 41.6 · 10^-5^ | 21.9 · 10^-5^ |  |  |  |
| tNAA | Mean | 458.2 · 10^-5^ | 482.1 · 10^-5^ | 345.4 · 10^-5^ | 1.000 | **0.017** | **0.014** |
|  | SD | 108.1 · 10^-5^ | 171.5 · 10^-5^ | 78.5 · 10^-5^ |  |  |  |
| tCr | Mean | 278.4 · 10^-5^ | 293.9 · 10^-5^ | 281.3 · 10^-5^ | 0.420 | 1.000 | 0.882 |
|  | SD | 61.8 · 10^-5^ | 93.1 · 10^-5^ | 44.4 · 10^-5^ |  |  |  |
| Glx | Mean | 424.0 · 10^-5^ | 370.4 · 10^-5^ | 393.9 · 10^-5^ | 1.000 | 0.869 | 1.000 |
|  | SD | 174.7 · 10^-5^ | 190.6 · 10^-5^ | 62.3 · 10^-5^ |  |  |  |

**Table S1d** Prefrontal cortex.

| **Metabolites** | | **HC** | | **SCA14** | | **SCA1** | | **P-value**  **SCA14-HC** | | **P-value**  **SCA1-HC** | | **P-value**  **SCA14-SCA1** |
| --- | --- | --- | --- | --- | --- | --- | --- | --- | --- | --- | --- | --- |
| Ala | Mean | 7.6 · 10^-5^ | 40.7 · 10^-5^ | | 23.1 · 10^-5^ | | 0.428 | | 0.751 | | 1.000 | |
|  | SD | 17.1 · 10^-5^ | 83.7 · 10^-5^ | | 38.0 · 10^-5^ | |  |  |  |  |  |  |
| Asp | Mean | 23.5 · 10^-5^ | 33.8 · 10^-5^ | | 54.0 · 10^-5^ | | 1.000 | | **0.025** | | 0.309 | |
|  | SD | 30.4 · 10^-5^ | 33.6 · 10^-5^ | | 27.7 · 10^-5^ | |  |  |  |  |  |  |
| Cr | Mean | 258.8 · 10^-5^ | 251.3 · 10^-5^ | | 242.8 · 10^-5^ | | 1.000 | | 1.000 | | 1.000 | |
|  | SD | 83.0 · 10^-5^ | 75.3 · 10^-5^ | | 81.4 · 10^-5^ | |  |  |  |  |  |  |
| PCr | Mean | 108.1 · 10^-5^ | 127.0 · 10^-5^ | | 117.4 · 10^-5^ | | 1.000 | | 1.000 | | 1.000 | |
|  | SD | 61.9 · 10^-5^ | 79.8 · 10^-5^ | | 76.7 · 10^-5^ | |  |  |  |  |  |  |
| GABA | Mean | 71.3 · 10^-5^ | 62.6 · 10^-5^ | | 50.3 · 10^-5^ | | 1.000 | | 0.396 | | 1.000 | |
|  | SD | 24.2 · 10^-5^ | 30.1 · 10^-5^ | | 33.9 · 10^-5^ | |  |  |  |  |  |  |
| Glc | Mean | 35.2 · 10^-5^ | 50.6 · 10^-5^ | | 5.3 · 10^-5^ | | 1.000 | | 0.429 | | 1.000 | |
|  | SD | 30.1 · 10^-5^ | 42.0 · 10^-5^ | | 30.9 · 10^-5^ | |  |  |  |  |  |  |
| Gln | Mean | 276.8 · 10^-5^ | 292.9 · 10^-5^ | | 283.5 · 10^-5^ | | 0.636 | | 1.000 | | 1.000 | |
|  | SD | 49.7 · 10^-5^ | 65.2 · 10^-5^ | | 52.0 · 10^-5^ | |  |  |  |  |  |  |
| Glu | Mean | 455.3 · 10^-5^ | 447.4 · 10^-5^ | | 413.6 · 10^-5^ | | 1.000 | | 0.483 | | 0.625 | |
|  | SD | 72.6 · 10^-5^ | 63.1 · 10^-5^ | | 50.4 · 10^-5^ | |  |  |  |  |  |  |
| GPC | Mean | 94.1 · 10^-5^ | 75.1 · 10^-5^ | | 84.4 · 10^-5^ | | 0.402 | | 0.668 | | 1.000 | |
|  | SD | 22.7 · 10^-5^ | 45.3 · 10^-5^ | | 16.9 · 10^-5^ | |  |  |  |  |  |  |
| PCh | Mean | 5.3 · 10^-5^ | 24.6 · 10^-5^ | | 7.4 · 10^-5^ | | 0.125 | | 1.000 | | 0.573 | |
|  | SD | 13.9 · 10^-5^ | 33.3 · 10^-5^ | | 14.7 · 10^-5^ | |  |  |  |  |  |  |
| GSH | Mean | 52.5 · 10^-5^ | 38.6 · 10^-5^ | | 49.8 · 10^-5^ | | 0.223 | | 1.000 | | 0.784 | |
|  | SD | 23.8 · 10^-5^ | 33.3 · 10^-5^ | | 14.9 · 10^-5^ | |  |  |  |  |  |  |
| Ins | Mean | 260.3 · 10^-5^ | 265.3 · 10^-5^ | | 259.7 · 10^-5^ | | 1.000 | | 1.000 | | 1.000 | |
|  | SD | 64.9 · 10^-5^ | 74.1 · 10^-5^ | | 21.7 · 10^-5^ | |  |  |  |  |  |  |
| Lac | Mean | 34.9 · 10^-5^ | 37.6 · 10^-5^ | | 41.0 · 10^-5^ | | 1.000 | | 1.000 | | 1.000 | |
|  | SD | 28.1 · 10^-5^ | 37.5 · 10^-5^ | | 34.4 · 10^-5^ | |  |  |  |  |  |  |
| NAA | Mean | 402.6 · 10^-5^ | 409.6 · 10^-5^ | | 370.2 · 10^-5^ | | 1.000 | | 0.510 | | 0.282 | |
|  | SD | 78.4 · 10^-5^ | 93.5 · 10^-5^ | | 59.6 · 10^-5^ | |  |  |  |  |  |  |
| NAAG | Mean | 47.7 · 10^-5^ | 50.4 · 10^-5^ | | 47.7 · 10^-5^ | | 1.000 | | 1.000 | | 1.000 | |
|  | SD | 53.6 · 10^-5^ | 82.7 · 10^-5^ | | 37.8 · 10^-5^ | |  |  |  |  |  |  |
| Scyllo | Mean | 7.4 · 10^-5^ | 6.6 · 10^-5^ | | 2.9 · 10^-5^ | | 1.000 | | 0.780 | | 1.000 | |
|  | SD | 8.8 · 10^-5^ | 8.5 · 10^-5^ | | 4.2 · 10^-5^ | |  |  |  |  |  |  |
| Tau | Mean | 17.3 · 10^-5^ | 24.5 · 10^-5^ | | 18.8 · 10^-5^ | | 1.000 | | 1.000 | | 1.000 | |
|  | SD | 24.6 · 10^-5^ | 34.2 · 10^-5^ | | 21.2 · 10^-5^ | |  |  |  |  |  |  |
| CrCH2 | Mean | 9.9 · 10^-5^ | 8.0 · 10^-5^ | | 3.5 · 10^-5^ | | 1.000 | | 1.000 | | 1.000 | |
|  | SD | 26.9 · 10^-5^ | 16.0 · 10^-5^ | | 7.6 · 10^-5^ | |  |  |  |  |  |  |
| tCho | Mean | 99.5 · 10^-5^ | 99.7 · 10^-5^ | | 91.7 · 10^-5^ | | 1.000 | | 0.915 | | 1.000 | |
|  | SD | 17.0 · 10^-5^ | 22.7 · 10^-5^ | | 8.5 · 10^-5^ | |  |  |  |  |  |  |
| tNAA | Mean | 450.3 · 10^-5^ | 459.9 · 10^-5^ | | 417.9 · 10^-5^ | | 1.000 | | 0.609 | | 0.369 | |
|  | SD | 70.4 · 10^-5^ | 53.3 · 10^-5^ | | 64.2 · 10^-5^ | |  |  |  |  |  |  |
| tCr | Mean | 367.0 · 10^-5^ | 378.3 · 10^-5^ | | 360.2 · 10^-5^ | | 1.000 | | 1.000 | | 1.000 | |
|  | SD | 62.0 · 10^-5^ | 61.2 · 10^-5^ | | 39.1 · 10^-5^ | |  |  |  |  |  |  |
| Glx | Mean | 732.3 · 10^-5^ | 740.3 · 10^-5^ | | 696.9 · 10^-5^ | | 1.000 | | 1.000 | | 0.832 | |
|  | SD | 101.6 · 10^-5^ | 88.9 · 10^-5^ | | 84.6 · 10^-5^ | |  |  |  |  |  |  |

**Table S1e** Motor cortex.

| **Metabolites** | | **HC** | **SCA14** | **SCA1** | **P-value**  **SCA14-HC** | **P-value**  **SCA1-HC** | **P-value**  **SCA14-SCA1** |
| --- | --- | --- | --- | --- | --- | --- | --- |
| Ala | Mean | 9.2 · 10^-5^ | 9.3 · 10^-5^ | 38.7 · 10^-5^ | 1.000 | 0.201 | 0.167 |
|  | SD | 14.9 · 10^-5^ | 16.8 · 10^-5^ | 51.2 · 10^-5^ |  |  |  |
| Asp | Mean | 43.1 · 10^-5^ | 29.3 · 10^-5^ | 31.1 · 10^-5^ | 0.163 | 0.600 | 1.000 |
|  | SD | 26.7 · 10^-5^ | 21.4 · 10^-5^ | 25.8 · 10^-5^ |  |  |  |
| Cr | Mean | 186.7 · 10^-5^ | 184.4 · 10^-5^ | 191.0 · 10^-5^ | 1.000 | 1.000 | 1.000 |
|  | SD | 64.1 · 10^-5^ | 47.1 · 10^-5^ | 55.6 · 10^-5^ |  |  |  |
| PCr | Mean | 143.4 · 10^-5^ | 127.4 · 10^-5^ | 107.3 · 10^-5^ | 1.000 | 0.072 | 0.431 |
|  | SD | 63.0 · 10^-5^ | 51.9 · 10^-5^ | 30.9 · 10^-5^ |  |  |  |
| GABA | Mean | 44.3 · 10^-5^ | 42.9 · 10^-5^ | 33.0 · 10^-5^ | 1.000 | 0.487 | 1.000 |
|  | SD | 24.1 · 10^-5^ | 28.4 · 10^-5^ | 27.8 · 10^-5^ |  |  |  |
| Glc | Mean | 46.5 · 10^-5^ | 59.6 · 10^-5^ | 55.4 · 10^-5^ | 0.739 | 0.871 | 1.000 |
|  | SD | 26.5 · 10^-5^ | 32.5 · 10^-5^ | 15.6 · 10^-5^ |  |  |  |
| Gln | Mean | 212.2 · 10^-5^ | 190.8 · 10^-5^ | 202.8 · 10^-5^ | 0.374 | 1.000 | 1.000 |
|  | SD | 42.3 · 10^-5^ | 56.1 · 10^-5^ | 36.9 · 10^-5^ |  |  |  |
| Glu | Mean | 416.9 · 10^-5^ | 383.9 · 10^-5^ | 373.3 · 10^-5^ | 0.119 | 0.147 | 1.000 |
|  | SD | 50.3 · 10^-5^ | 69.8 · 10^-5^ | 70.4 · 10^-5^ |  |  |  |
| GPC | Mean | 74.8 · 10^-5^ | 73.7 · 10^-5^ | 68.7 · 10^-5^ | 1.000 | 0.936 | 1.000 |
|  | SD | 15.6 · 10^-5^ | 15.3 · 10^-5^ | 14.0 · 10^-5^ |  |  |  |
| PCh | Mean | 2.7 · 10^-5^ | 2.6 · 10^-5^ | <0.1 · 10^-5^ | 1.000 | 0.416 | 0.962 |
|  | SD | 7.3 · 10^-5^ | 8.1 · 10^-5^ | <0.1 · 10^-5^ |  |  |  |
| GSH | Mean | 47.2 · 10^-5^ | 43.4 · 10^-5^ | 46.1 · 10^-5^ | 1.000 | 1.000 | 1.000 |
|  | SD | 21.1 · 10^-5^ | 14.4 · 10^-5^ | 22.1 · 10^-5^ |  |  |  |
| Ins | Mean | 233.9 · 10^-5^ | 226.7 · 10^-5^ | 199.8 · 10^-5^ | 1.000 | 0.097 | 0.574 |
|  | SD | 33.0 · 10^-5^ | 52.0 · 10^-5^ | 47.3 · 10^-5^ |  |  |  |
| Lac | Mean | 31.3 · 10^-5^ | 40.9 · 10^-5^ | 44.6 · 10^-5^ | 1.000 | 1.000 | 1.000 |
|  | SD | 24.2 · 10^-5^ | 34.4 · 10^-5^ | 45.2 · 10^-5^ |  |  |  |
| NAA | Mean | 401.8 · 10^-5^ | 374.8 · 10^-5^ | 338.3 · 10^-5^ | 0.370 | **0.015** | 0.611 |
|  | SD | 66.0 · 10^-5^ | 71.4 · 10^-5^ | 55.1 · 10^-5^ |  |  |  |
| NAAG | Mean | 60.1 · 10^-5^ | 55.2 · 10^-5^ | 73.8 · 10^-5^ | 1.000 | 1.000 | 0.491 |
|  | SD | 26.9 · 10^-5^ | 51.9 · 10^-5^ | 34.8 · 10^-5^ |  |  |  |
| Scyllo | Mean | 4.8 · 10^-5^ | 4.3 · 10^-5^ | 0.5 · 10^-5^ | 1.000 | **0.003** | **0.039** |
|  | SD | 5.8 · 10^-5^ | 4.9 · 10^-5^ | 1.2 · 10^-5^ |  |  |  |
| Tau | Mean | 13.6 · 10^-5^ | 23.1 · 10^-5^ | 2.5 · 10^-5^ | 0.913 | 0.263 | 0.055 |
|  | SD | 19.1 · 10^-5^ | 27.5 · 10^-5^ | 4.7 · 10^-5^ |  |  |  |
| CrCH2 | Mean | 3.0 · 10^-5^ | 10.7 · 10^-5^ | <0.1 · 10^-5^ | 0.297 | 0.894 | 0.070 |
|  | SD | 10.2 · 10^-5^ | 18.4 · 10^-5^ | <0.1 · 10^-5^ |  |  |  |
| tCho | Mean | 77.5 · 10^-5^ | 76.3 · 10^-5^ | 68.7 · 10^-5^ | 1.000 | 0.339 | 0.924 |
|  | SD | 13.6 · 10^-5^ | 13.6 · 10^-5^ | 14.0 · 10^-5^ |  |  |  |
| tNAA | Mean | 461.9 · 10^-5^ | 429.9 · 10^-5^ | 412.2 · 10^-5^ | 0.316 | 0.082 | 1.000 |
|  | SD | 68.2 · 10^-5^ | 72.9 · 10^-5^ | 72.4 · 10^-5^ |  |  |  |
| tCr | Mean | 330.2 · 10^-5^ | 311.8 · 10^-5^ | 298.3 · 10^-5^ | 0.412 | 0.225 | 1.000 |
|  | SD | 48.6 · 10^-5^ | 47.3 · 10^-5^ | 53.7 · 10^-5^ |  |  |  |
| Glx | Mean | 629.2 · 10^-5^ | 574.8 · 10^-5^ | 576.3 · 10^-5^ | 0.261 | 0.344 | 1.000 |
|  | SD | 73.0 · 10^-5^ | 101.6 · 10^-5^ | 100.0 · 10^-5^ |  |  |  |

Abbreviations: HC, healthy controls; SCA14, spinocerebellar ataxia type 14; SCA1, spinocerebellar ataxia type 1; SD, standard deviation; AU, arbitrary units; VOI, volume of interest; Ala, alanine; Asp, aspartate; Cr, creatine; PCr, phosphocreatine; GABA, γ-aminobutyric acid; Glc, glucose; Gln, glutamine; Glu, glutamate; GPC, glycerophosphocholine; PCh, phosphocholine; GSH, glutathione; Ins, myo-inositol; Lac, lactate; NAA, n-acetylaspartate; NAAG, n-acetylaspartylglutamate; Scyllo, scyllo-inositol; Tau, taurine; CrCH2, negative creatine methylene; tCho, total choline (GPC+PCh); tNAA, total n-acetylaspartate (NAA+NAAG); tCr, total creatine (Cr+PCr); Glx, glutamate+glutamine.

**Table S2** Multivariable linear regression analysis of clinical parameters (SARA, DemTect, age of onset, and disease duration) and metabolite levels with adjustment for age in a merged patient group of SCA14 and SCA1. Unstandardized regression coefficients (B) and p-values are given for each VOI and metabolite.

| **Metabolites** | | **SARA** | | **DemTect** | | **Age of onset** | | **Disease duration** | |
| --- | --- | --- | --- | --- | --- | --- | --- | --- | --- |
|  |  | **B** | **P-value** | **B** | **P-value** | **B** | **P-value** | **B** | **P-value** |
| Ala | Vermis | -1730.9 | 0.356 | 1119.0 | 0.586 | -4454.4 | 0.319 | 4371.5 | 0.326 |
|  | Cerebellar hemisphere | -2863.7 | 0.417 | 2251.3 | 0.241 | -8381.9 | 0.239 | 8476.4 | 0.231 |
|  | Pons | 23.1 | 0.994 | -3688.3 | 0.162 | 873.7 | 0.929 | -873.7 | 0.929 |
|  | Prefrontal cortex | 236.8 | 0.922 | 88.1 | 0.987 | -5540.4 | 0.174 | 5476.8 | 0.176 |
|  | Motor cortex | 1352.0 | 0.672 | 1910.0 | 0.272 | 8646.2 | 0.158 | -8706.4 | 0.153 |
| Asp | Vermis | -3942.5 | 0.185 | 1015.1 | 0.764 | 2675.4 | 0.713 | -2645.9 | 0.714 |
|  | Cerebellar hemisphere | 2733.9 | 0.564 | 2298.9 | 0.372 | -6483.7 | 0.501 | 6327.9 | 0.509 |
|  | Pons | -2809.6 | 0.128 | 2241.4 | 0.194 | -3460.9 | 0.440 | 3460.9 | 0.440 |
|  | Prefrontal cortex | 10966.0 | **0.005** | -74,3 | 0.979 | -8920.0 | 0.227 | 8924.9 | 0.223 |
|  | Motor cortex | -1152.3 | 0.821 | 1601.1 | 0.575 | -10136.7 | 0.302 | 10479.3 | 0.284 |
| Cr | Vermis | 85.6 | 0.900 | 360.7 | 0.583 | 1054.7 | 0.514 | -1028.2 | 0.522 |
|  | Cerebellar hemisphere | 588.9 | 0.676 | 105.0 | 0.891 | 4628.8 | 0.096 | -4560.7 | 0.099 |
|  | Pons | -898.2 | 0.404 | 132.7 | 0.896 | 1832.2 | 0.503 | -1832.2 | 0.503 |
|  | Prefrontal cortex | -128.1 | 0.948 | -1877.4 | 0.072 | -3400.1 | 0.334 | 3358.0 | 0.336 |
|  | Motor cortex | -2478.2 | 0.328 | 1595.3 | 0.248 | -6087.0 | 0.216 | 6025.8 | 0.219 |
| PCr | Vermis | 825.7 | 0.391 | -1277.8 | 0.148 | 137.3 | 0.953 | -156.8 | 0.946 |
|  | Cerebellar hemisphere | -936.9 | 0.521 | 160.8 | 0.842 | -1415.1 | 0.635 | 1368.1 | 0.644 |
|  | Pons | -50.3 | 0.963 | 432.2 | 0.681 | -2111.5 | 0.444 | 2111.5 | 0.444 |
|  | Prefrontal cortex | 942.5 | 0.598 | 1781.3 | 0.093 | 2380.8 | 0.471 | -2346.4 | 0.475 |
|  | Motor cortex | 1680.3 | 0.528 | -41.1 | 0.978 | -613.0 | 0.907 | 705.2 | 0.892 |

| GABA | Vermis | -4280.9 | 0.160 | 4394.6 | 0.103 | 6226.8 | 0.424 | -6030.2 | 0.436 |
| --- | --- | --- | --- | --- | --- | --- | --- | --- | --- |
|  | Cerebellar hemisphere | 646.0 | 0.889 | 1054.7 | 0.680 | 8124.2 | 0.385 | -8082.2 | 0.386 |
|  | Pons | -3091.5 | 0.438 | 5082.6 | 0.173 | 5263.2 | 0.584 | -5263.2 | 0.584 |
|  | Prefrontal cortex | -6630.5 | 0.132 | 3851.3 | 0.143 | 4244.5 | 0.629 | -4092.7 | 0.639 |
|  | Motor cortex | -2687.0 | 0.527 | 722.4 | 0.756 | -4452.7 | 0.592 | 4437.9 | 0.592 |
| Glc | Vermis | 1727.3 | 0.354 | -940.3 | 0.635 | -343.4 | 0.939 | 366.8 | 0.935 |
|  | Cerebellar hemisphere | 305.4 | 0.903 | 1606.5 | 0.232 | 4595.8 | 0.364 | -4547.7 | 0.367 |
|  | Pons | -289.9 | 0.909 | -1640.5 | 0.477 | -11880.8 | **0.042** | 11880.8 | **0.042** |
|  | Prefrontal cortex | 2787.5 | 0.459 | -4285.9 | 0.073 | -1870.6 | 0.792 | 1776.5 | 0.801 |
|  | Motor cortex | -625.5 | 0.888 | 3184.7 | 0.234 | -3930.3 | 0.649 | 3844.9 | 0.654 |
| Gln | Vermis | -215.7 | 0.895 | 242.2 | 0.883 | 1064.3 | 0.795 | -911.9 | 0.823 |
|  | Cerebellar hemisphere | -1054.8 | 0.559 | -1291.6 | 0.177 | 4420.2 | 0.222 | -4406.2 | 0.221 |
|  | Pons | 914.2 | 0.707 | -3727.6 | 0.198 | 9140.6 | 0.099 | -9140.6 | 0.099 |
|  | Prefrontal cortex | 2464.2 | 0.286 | 1006.1 | 0.450 | -518.9 | 0.899 | 448.5 | 0.912 |
|  | Motor cortex | -2095.4 | 0.380 | 2821.5 | **0.024** | -3139.2 | 0.502 | 3258.7 | 0.484 |
| Glu | Vermis | 1023.7 | 0.305 | -1007.8 | 0.266 | 1013.3 | 0.674 | -1009.9 | 0.673 |
|  | Cerebellar hemisphere | -2339.9 | 0.227 | 1555.7 | 0.163 | 1905.9 | 0.634 | -1951.8 | 0.624 |
|  | Pons | -1822.9 | 0.172 | 1685.4 | 0.272 | 2004.2 | 0.534 | -2004.2 | 0.534 |
|  | Prefrontal cortex | -787.3 | 0.759 | 650.4 | 0.672 | 779.4 | 0.861 | -837.8 | 0.850 |
|  | Motor cortex | -1927.6 | 0.297 | 876.9 | 0.383 | -1787.0 | 0.624 | 1828.3 | 0.614 |
| GPC | Vermis | -458.4 | 0.918 | 177.5 | 0.968 | 9610.8 | 0.361 | -950.3.6 | 0.364 |
|  | Cerebellar hemisphere | -8216.8 | **0.043** | 5749.6 | **0.006** | 4913.6 | 0.572 | -4793.1 | 0.579 |
|  | Pons | -4827.6 | 0.084 | 2406.3 | 0.378 | -7680.1 | 0.260 | 7680.1 | 0.260 |
|  | Prefrontal cortex | 1915.6 | 0.645 | 1523.8 | 0.552 | 6813.9 | 0.342 | -6813.6 | 0.338 |
|  | Motor cortex | 1588.2 | 0.869 | 5739.8 | 0.288 | -30544.0 | 0.094 | 30334.4 | 0.095 |
| PCh | Vermis | -4126.4 | 0.776 | 8161.9 | 0.526 | -102989.3 | **0.001** | 102791.8 | **0.001** |
|  | Cerebellar hemisphere | 2567.8 | 0.639 | -6475.2 | **0.019** | 8502.1 | 0.443 | -8576.1 | 0.437 |
|  | Pons | -73.0 | 0.985 | 1550.3 | 0.696 | 5715.6 | 0.518 | -5715.6 | 0.518 |
|  | Prefrontal cortex | -1895.5 | 0.708 | -2348.0 | 0.428 | -9554.0 | 0.274 | 9516.4 | 0.272 |
|  | Motor cortex | -21082.8 | 0.266 | 10655.4 | 0.299 | -4632.5 | 0.902 | 4511.9 | 0.904 |
| GSH | Vermis | 1653.1 | 0.403 | -1323.2 | 0.514 | 5552.9 | 0.233 | -5510.9 | 0.234 |
|  | Cerebellar hemisphere | -1091.5 | 0.748 | 704.8 | 0.715 | 8560.0 | 0.207 | -8414.2 | 0.213 |
|  | Pons | -3515.5 | 0.341 | -1676.3 | 0.641 | 2135.9 | 0.835 | -2135.9 | 0.835 |
|  | Prefrontal cortex | 3979.9 | 0.451 | -1270.0 | 0.736 | -6292.5 | 0.495 | 6282.2 | 0.492 |
|  | Motor cortex | 1356.5 | 0.844 | 2089.7 | 0.581 | 3084,3 | 0.819 | -3165.4 | 0.814 |
| Ins | Vermis | 1737.4 | 0.124 | -1117.5 | 0.354 | 2869.4 | 0.289 | -2830.0 | 0.293 |
|  | Cerebellar hemisphere | 803.2 | 0.608 | -183.1 | 0.832 | 5973.2 | **0.050** | -5837.2 | 0.055 |
|  | Pons | -350.5 | 0.848 | -2585.9 | 0.161 | 5499.6 | 0.252 | -5499.6 | 0.252 |
|  | Prefrontal cortex | 1621.5 | 0.560 | 387.8 | 0.875 | -2961.5 | 0.540 | 2958.5 | 0.537 |
|  | Motor cortex | -344.5 | 0.896 | 1217.2 | 0.424 | -8942.4 | 0.071 | 8899.2 | 0.071 |
| Lac | Vermis | -991.9 | 0.619 | -29.2 | 0.987 | 3045.3 | 0.577 | -2980.1 | 0.583 |
|  | Cerebellar hemisphere | -4241.9 | 0.244 | 3263.6 | 0.099 | -7405.5 | 0.321 | 7252.0 | 0.329 |
|  | Pons | 585.0 | 0.875 | 126.5 | 0.972 | 6507.6 | 0.485 | -6507.6 | 0.485 |
|  | Prefrontal cortex | 5479.8 | 0.229 | -64.0 | 0.983 | 9122.3 | 0.264 | -9376.2 | 0.246 |
|  | Motor cortex | 5693.2 | 0.103 | 1003.4 | 0.629 | 5566.5 | 0.425 | -5534.7 | 0.426 |
| NAA | Vermis | 397.4 | 0.744 | -370.3 | 0.748 | 1060.8 | 0.712 | -1054.5 | 0.712 |
|  | Cerebellar hemisphere | -3674.9 | **0.048** | 1207.4 | 0.255 | 3582.9 | 0.363 | -3572.1 | 0.363 |
|  | Pons | -1826.1 | 0.055 | 1846.8 | 0.064 | -812.8 | 0.756 | 812.8 | 0.756 |
|  | Prefrontal cortex | -2277.1 | 0.208 | 529,2 | 0.664 | 789.7 | 0.812 | -710.5 | 0.829 |
|  | Motor cortex | -236.9 | 0.905 | 1198.5 | 0.298 | -8783.1 | **0.016** | 8801.6 | **0.016** |
| NAAG | Vermis | -870.2 | 0.523 | 1115.3 | 0.411 | 274.9 | 0.933 | -228.9 | 0.944 |
|  | Cerebellar hemisphere | -2222.5 | 0.293 | 2888.2 | **0.016** | -5166.3 | 0.228 | 5105.2 | 0.232 |
|  | Pons | -853.0 | 0.538 | 619.6 | 0.709 | -3690.7 | 0.253 | 3690.7 | 0.253 |
|  | Prefrontal cortex | 3539.8 | 0.114 | -462.8 | 0.742 | -894.1 | 0.824 | 808.1 | 0.840 |
|  | Motor cortex | -3500.4 | 0.166 | -148.9 | 0.919 | 11888.2 | **0.012** | -11854.5 | **0.012** |
| Scyllo | Vermis | 1677.4 | 0.877 | -1753.7 | 0.868 | 3913.3 | 0.879 | -3750.8 | 0.883 |
|  | Cerebellar hemisphere | -6717.0 | 0.553 | -4834.0 | 0.439 | 35924.1 | 0.109 | -36000.5 | 0.106 |
|  | Pons | -4812.2 | 0.744 | 3254.6 | 0.845 | -34698.2 | 0.322 | 34698.2 | 0.322 |
|  | Prefrontal cortex | -12712.3 | 0.556 | 242.6 | 0.988 | -10888.2 | 0.772 | 10220.3 | 0.785 |
|  | Motor cortex | -20889.4 | 0.481 | 12466.5 | 0.460 | -70676.7 | 0.217 | 69879.6 | 0.221 |
| Tau | Vermis | -247.8 | 0.909 | -419.6 | 0.833 | 6515.2 | 0.200 | -6607.3 | 0.191 |
|  | Cerebellar hemisphere | -287.8 | 0.947 | -3732.2 | 0.102 | 11667.1 | 0.173 | -11346.0 | 0.184 |
|  | Pons | -484.1 | 0.852 | -196.8 | 0.958 | 2662.0 | 0.663 | -2662.0 | 0.663 |
|  | Prefrontal cortex | -6064.5 | 0.208 | 4225.0 | 0.294 | -3892.2 | 0.649 | 3909.5 | 0.645 |
|  | Motor cortex | -6002.4 | 0.228 | 1124.6 | 0.724 | -12805.1 | 0.187 | 12745.8 | 0.187 |
| CrCH2 | Vermis | 2342.3 | 0.098 | -1612.9 | 0.209 | 2691.5 | 0.434 | -2721.1 | 0.427 |
|  | Cerebellar hemisphere | -1564.9 | 0.682 | -2798.6 | 0.165 | 6515.8 | 0.399 | -6553.4 | 0.394 |
|  | Pons | -92.9 | 0.944 | 460.9 | 0.776 | 1682.8 | 0.586 | -1682.8 | 0.586 |
|  | Prefrontal cortex | -10841.5 | 0.310 | -3337.1 | 0.699 | 19110.6 | 0.305 | -18616.7 | 0.314 |
|  | Motor cortex | -269.9 | 0.973 | 3841.9 | 0.453 | -10785.8 | 0.489 | 10662.5 | 0.492 |
| tCho | Vermis | -889.7 | 0.846 | 1234.3 | 0.790 | -177.7 | 0.987 | 271.5 | 0.980 |
|  | Cerebellar hemisphere | -16674.3 | **0.007** | 4537.9 | 0.218 | 25377.8 | 0.054 | -25189.0 | 0.055 |
|  | Pons | -8530.7 | **0.015** | 8025.9 | **0.047** | -7802.6 | 0.409 | 7802.6 | 0.409 |
|  | Prefrontal cortex | 2627.9 | 0.758 | -1278.9 | 0.837 | 1834.2 | 0.902 | -1938.7 | 0.896 |
|  | Motor cortex | -4068.4 | 0.686 | 9744.0 | 0.081 | -34855.2 | 0.067 | 34591.6 | 0.068 |
| tNAA | Vermis | -363.5 | 0.791 | 494.4 | 0.695 | 1753.3 | 0.601 | -1695.8 | 0.611 |
|  | Cerebellar hemisphere | -3854.6 | **0.011** | 2271.6 | **0.006** | -458.0 | 0.891 | 430.2 | 0.897 |
|  | Pons | -1828.3 | **0.028** | 1625.1 | 0.063 | -2228.7 | 0.304 | 2228.7 | 0.304 |
|  | Prefrontal cortex | -75,2 | 0.978 | 392.8 | 0.825 | 421.0 | 0.935 | -370.6 | 0.942 |
|  | Motor cortex | -2069.8 | 0.267 | 949.0 | 0.377 | -1561.8 | 0.672 | 1596.9 | 0.663 |
| tCr | Vermis | 1025.7 | 0.291 | -614.1 | 0.504 | 2302.2 | 0.317 | -2267.4 | 0.322 |
|  | Cerebellar hemisphere | -460.7 | 0.797 | 429.9 | 0.665 | 5292.3 | 0.135 | -5253.4 | 0.136 |
|  | Pons | -3019.3 | 0.102 | 3837.9 | 0.139 | -605.2 | 0.895 | 605.2 | 0.895 |
|  | Prefrontal cortex | 2693,5 | 0.405 | -990.7 | 0.747 | -1658.9 | 0.771 | 1654.9 | 0.770 |
|  | Motor cortex | -1244.8 | 0.675 | 2371.8 | 0.159 | -9044.2 | 0.110 | 9075.6 | 0.107 |
| Glx | Vermis | 587.5 | 0.462 | -595.8 | 0.414 | 881.8 | 0.643 | -846.5 | 0.655 |
|  | Cerebellar hemisphere | -1304.8 | 0.266 | -66.4 | 0.919 | 2615.6 | 0.273 | -2626.2 | 0.269 |
|  | Pons | -1011.2 | 0.360 | 599.7 | 0.412 | 3219.9 | 0.210 | -3219.9 | 0.210 |
|  | Prefrontal cortex | 873,1 | 0.591 | 685.3 | 0.448 | 69,4 | 0.980 | -126.8 | 0.964 |
|  | Motor cortex | -1406.0 | 0.249 | 1121.4 | 0.086 | -1589.5 | 0.509 | 1639.5 | 0.494 |

Abbreviations: VOI, volume of interest; SARA, scale for the assessment and rating of ataxia; DemTect, score of the cognitive screening test for mild cognitive impairment and early dementia; Ala, alanine; Asp, aspartate; Cr, creatine; PCr, phosphocreatine; GABA, γ-aminobutyric acid; Glc, glucose; Gln, glutamine; Glu, glutamate; GPC, glycerophosphocholine; PCh, phosphocholine; GSH, glutathione; Ins, myo-inositol; Lac, lactate; NAA, n-acetylaspartate; NAAG, n-acetylaspartylglutamate; Scyllo, scyllo-inositol; Tau, taurine; CrCH2, negative creatine methylene; tCho, total choline (GPC+PCh); tNAA, total n-acetylaspartate (NAA+NAAG); tCr, total creatine (Cr+PCr); Glx, glutamate+glutamine.
